# Research on residual GM optimization based on PEMEA-BP correction

**DOI:** 10.1038/s41598-020-77630-w

**Published:** 2020-12-09

**Authors:** Junhang Duan, Ling Zhu, Wei Xing, Xi Zhang, Zhong Peng, Huating Gou

**Affiliations:** 1 Department of Automation, Tsinghua University, Beijing, 10084 China; 2 College of Information System and Management, National University of Defense Technology, Changsha, 410073 China; 3 Beijing Foreign Studies University, Beijing, 100089 China; 4 Department of Automation, Hunan University, Changsha, 410073 China

**Keywords:** Computational science, Statistics

## Abstract

With the advantages of small samples and high accuracy, Grey Model (GM) still has two major problems need to be addressed, high input data requirements and large margin of error. Hence, this paper proposes an algorithm based on Populational Entropy Based Mind Evolutionary Algorithm-Error Back Propagation Training Artificial Neural Algorithm to modify GM residual tail, which will not only keep the advantages of GM, but also expand its scope of use to various non-linear and even multidimensional objects. Meanwhile, it can avoid defects of other algorithms, such as slow convergence and easy to fall into the local minimum. In small samples data experiments, judging from SSE, MAE, MSE, MAPE, MRE and other indicators, this new algorithm has significant advantage over GM, BP algorithm and combined genetic algorithm in terms of simulation accuracy and convergence speed.

## Introduction

The development of forecasting technology over the last decade contributed to the construction of approximately 150 forecasting methods, including the method based on market research analysis, Delphi method based on expert opinion, time series method based on similarity, regression analysis, grey prediction model and artificial neural network methods, etc. Among them, the grey prediction model is based on the grey systems theory, mainly designed to solve the uncertainty problems of “small samples” and “poor information” which are difficult for probability theory statistics and fuzzy mathematics. Grey systems theory use 3 colors, black, grey and white to describe the state of a system. According to the degree of its inaccuracy due to the lack of information, the system can be described from black through grey to white—from completely missed information to completely known information. Since in reality inaccuracy is inevitable, black and white situations seldom occur. Most situations fall somewhere in between, i.e., we have only partial information, which we call “grey”. Based on this understanding, the grey systems theory advocates that fragment information can also be used to speculate about the unknown information and the whole picture; it is not regarding the perfect speculation, it is concerning the development and patterns analysis and description of the system. It is similar to difference-differential equation model, which is compatible with difference, differential, index, and can be considered as a time-varying function. The biggest advantage of grey prediction model is that it does not require the support of a large amount of data, nor does it require the data to be subject to typical distribution to achieve better prediction results.

### Literature and review

Debnath and Mourshed^1^ has summarized 50 different prediction methodsin their previous study, they found that artificial neural network (ANN) model was the most widely used one, and the second was support vector machine (SVM), followed by autoregressive integrated moving average (ARIMA) method, fuzzy logic (FL), linear regression (LR) and GM. However, predicted results of these single models are not as ideal as those of the mixed mode^2^. Rana et al.^3^ in their research proposed a way of two-dimensional (2D) interval forecasts. Their algorithm uses support vector regression method to carry out 2D interval forecasting solar energy. Currently, a considerable amount of researches were conducted on grey models, but primarily focus on the optimization of the model, the improvement of its simulation and prediction effects. The grey prediction model has developed rapidly, it has been widely used in prediction of pollutant, new energy industry^4^, electric power forecasting and petroleum production^5^. Wang et al.^6^ proposed a GM based on buffer operators. He used the buffer operator to identify the structural variation data. GA has been used to optimize the parameters. The method efficiently elevated the prediction accuracy. Sun et al.^7^ put forward a new algorithm using weighted Markov chain and grey model to obtain seasonal index of data. This algorithm aims to gain high-accuracy prediction in energy generation. Zheng^8^ utilized the Monte-Carlo simulation method to compare the discrete grey model (DGM) and traditional GM, finding that the predictive capabilities of the two models for random sequences conforming to normal distribution are nearly equivalent. However, the predictive capabilities of DGM for the other aspects of random sequences are all superior to those of GM. Ma et al.^9^ proposed a fractional time delayed grey model to solve the time delayed effect and improve fractional GM. In order to optimize the value and grey derivatives, Tan^10^ improved background value and redefined the solution formula for background value. Bo and Wei^11^ established a new GM with a higher prediction capability by using the weighted mean of forward and backward difference quotients as the whitening value of grey derivative of GM model. Yuan et al.^12^ established a hybrid model by combining autoregressive integrated moving average (ARIMA) model with GM model to predict the energy consumption of China. Gatabazi et al.^13^ combined the grey model and Lotka–Volterra model to assess the interaction between cryptocurrencies. Zeng et al.^14^ established a new grey dynamic prediction model, a linear correction term, and a random disturbance term to the traditional GM (1, N) model to improve the prediction accuracy. In order to predict the non-equidistant sequences with integral range or digital range, Yao et al.^15^ proposed a generalized discrete grey model. By adopting background value optimization and central-point triangular whitenization weight function in GM and Markov chain, Ye et al.^16^ built an improved Grey–Markov forecasting model. Jeffrey Ofosu-Adarkwa^17^ develops an interval grey number-based approach to calculate the relative uncertainty. The proposed model, V-GM (1, N), is found to give the high accuracy in simulating the actual cement industry CO2 emissions data from 2005 to 2018. Xu^18^ proposed a new forecasting method to forecast greenhouse gas emissions in China from 2020 to 2025. Their model was a combination of the rolling grey prediction model and a buffered rolling method. Also there are other research outcomes such as Luo Youxin’s research on non-equal spacing sequence modeling, Liu Sifeng’s^19^ on the basic form and scope of GM (1, 1), Salmeron’s, Jose L., and Zhang Qishan’s on the improvement of model accuracy through combining the grey systems and other soft computing methods, Wang Zhengxin’s^20^ on the periodic GM (1,1) of power consumption, Wang Jianzhou’s^5^ on a multi-objective Ant Lion Optimizer to improve GM, Wang Qiang’s^21^ on a nonlinear dynamic GM to predicts oil consumption, and Zhang Q’s^22^ on metabolic GM and so on.

### Methods and innovations

Similar to other methods, the grey model method also bears the limitation of insufficient accuracy and large error. Hence, the issue of improving the accuracy on the premise of multiple types of input has received considerable critical attention from researchers dedicated to GM. An effective way to correct and improve the simulation accuracy, which is called residual grey model, is to set a model for actual sequence value ${x^{(0)}}(k)$ and the residual sequence ${\varepsilon ^{(0)}}(k)$ of analog sequence value ${{\hat{x}}^{(0)}}(k)$. Although the residual grey model simulation is known for its high accuracy, it is set under restricted conditions, that is, the tail segment eligible for modeling is linear with the same symbol. However, in reality, most sequences are in wave form or highly non-linear^23–25^. It is impossible to construct grey models for them to use the method of the residual tail. This paper proposes a new method based on Populational Entropy Based Mind Evolutionary Algorithm-Error Back Propagation Training Artificial Neural Algorithm (PEMEA-BP) to correct the residual tail segment to solve this problem. The basic idea is as follows: (a) Using the BP neural network to realize the mapping from N dimension to M dimension space and the ability to imitate multiple functions without having to assume that there is a certain relationship between data^26–28^. Take the residual tail as sample, enter it into the trained BP artificial neural network model, and obtain the effective residual sequence prediction value ${{\hat{\varepsilon }} ^{(0)}}(i)$; (b) To solve the problems that the BP artificial neural network is apt to fall into the local minimum and has low convergence speed, this new method adopt the Populational Entropy Based Mind Evolutionary Algorithm (PEMEA) to modify and output optimal connection value; (c) Obtain the final predicted value through residual grey model theory. The improved algorithm has the advantage of small GM residual model sample, high advantages of accuracy and the expanded the use scale which even covers the various nonlinear and multidimensional object. The practical application demonstrated that is algorithm is superior to other mainstream algorithms not only in analog accuracy but also in convergence speed.

In brief, this paper contributes in two aspects: (a) Our algorithm extends the use of the grey residual model, mainly expands the applicable scope of tail-segment data to include data with different sign and non-linear data. (b) We bring forward an algorithm to rectify the disruptive nature of BP via PEMEA, that is its tendency toward local optimality and slow convergence.

## Residual grey model

Grey model is among the most widely used model of the grey prediction theory, especially the mean value GM (1, 1) model proposed by Professor Deng Julong. When the mean G M (1, 1) modeled fail to meet the requirements, the residual model is adopted and amend it. The construction process of mean G M (1, 1) model is as follows:

1. Construct the 1-*AGO* sequence of *X* sequence^29^.

Set sequence $X^{(0)}= ({x^{(0)}}(1),{x^{(0)}}(2) \ldots ,{x^{(0)}}(n)),$ wherein ${x^{(0)}}(k) \ge 0,k = 1,2 \ldots ,n.$ And $X^{(1)}$ is the 1-*AGO* sequence of $X^{(0)}$: ${X^{(1)}} = ({x^{(1)}}(1),{x^{(1)}}(2)\ldots ,{x^{(1)}}(n))$, among which

1 $$\begin{aligned} {x^{(1)}}(k) = \sum \limits _{i = 1}^k {{x^{(0)}}(i),k = 1,\ldots n} \end{aligned}$$

2. Set the mean sequence ${Z^{(1)}}$ of the $1-AGO$ sequence

$$ {Z^{(1)}} = ({z^{(1)}}(2),{z^{(1)}}(3)\ldots ,{z^{(1)}}(n)) $$, among which

2 $$\begin{aligned} {z^{(1)}}(k) = \frac{1}{2}({x^{(1)}}(k) - ({x^{(1)}}(k - 1)) \end{aligned}$$

The above two steps are to “whiten” the existing data by constructing the 1-AGO sequence and the mean value sequence respectively, so as to fully explore its inherent laws and information.

3. Construct and solve the whitening differential equation.

Set ${x^{(0)}}(k) + a{z^{(1)}}(k) = b$ as the mean value form of G M (1, 1), hence the whitening differential equation would be:

3 $$\begin{aligned} \frac{{d{x^{(1)}}}}{{dt}} + a{x^{(1)}} = b \end{aligned}$$

Set the parameter vector as ${\hat{a}} = {(a,b)^T}$, therefore least squares method could be used to estimate

4 $$\begin{aligned} {\hat{a}}= & {} {({B^T}B)^{ - 1}}{B^T}Y \end{aligned}$$

5 $$\begin{aligned} Y= & {} \left[ {\begin{array}{*{20}{c}} {{x^{(0)}}(2)}\\ {{x^{(0)}}(3)}\\ \vdots \\ {{x^{(0)}}(n)} \end{array}} \right] ,B = \left[ {\begin{array}{*{20}{c}} { - {z^{(1)}}(2)}&{}\quad 1\\ { - {z^{(1)}}(3)}&{}\quad 1\\ \vdots &{}\quad \vdots \\ { - {z^{(1)}}(n)}&{}\quad 1 \end{array}} \right] \end{aligned}$$

4. Calculate the response time.

Parameter vector ${\hat{a}}$ could be calculated from (4) and (5), therein—a is development coefficient, which reflects the development trend of ${{\hat{x}}^{(1)}}$ and ${x^{(0)}}$, and *b* is the gray action. The time response of ${{\hat{x}}^{(1)}}(k)$ would be:

6 $$\begin{aligned} {{\hat{x}}^{(1)}}(k) = \left( {x^{(0)}}(1) - \frac{b}{a}\right) {e^{ - a(k - 1)}} + \frac{b}{a},k = 1,2 \ldots ,n \end{aligned}$$

From (6), the reduced reduction equation would be:

7 $$\begin{aligned} {{\hat{x}}^{(0)}}(k)= & {} {{\hat{x}}^{(1)}}(k) - {{\hat{x}}^{(1)}}(k - 1),k = 1,2\ldots ,n \end{aligned}$$

8 $$\begin{aligned} {{\hat{x}}^{(0)}}(k)= & {} (1 - {e^a})\left[ \left( {x^{(0)}}(1) - \frac{b}{a}\right) {e^{ - a(k - 1)}} \right] ,k = 1,2\ldots ,n \end{aligned}$$

And the analog sequence would be obtained from (8).

5. Calculate error.

The prediction results are evaluated by square sum error (SSE), mean absolute error (MAE), mean square error (MSE), mean absolute percentage error (MAPE), and mean relative error (MRE), as in Eqs. (9) to (13):

9 $$\begin{aligned} SSE= & {} \sum \limits _{i = 1}^n {{{({x^{(0)}}(i) - {{{\hat{x}}}^{(0)}}(i))}^2}} \end{aligned}$$

10 $$\begin{aligned} MAE= & {} \frac{1}{n}\sum \limits _{i = 1}^n {\left| {{x^{(0)}}(i) - {{{\hat{x}}}^{(0)}}(i)} \right| } \end{aligned}$$

11 $$\begin{aligned} MSE= & {} \frac{1}{n}\sum \limits _{i = 1}^n {{{({x^{(0)}}(i) - {{{\hat{x}}}^{(0)}}(i))}^2}} \end{aligned}$$

12 $$\begin{aligned} MAPE= & {} \frac{{\sum {_{i = 1}^n\left| {\frac{{{x^{(0)}}(i) - {{{\hat{x}}}^{(0)}}(i)}}{{{x^{(0)}}(i)}}} \right| } }}{n} \times 100\% \end{aligned}$$

13 $$\begin{aligned} MRE= & {} \frac{1}{n}\sum \limits _{i = 1}^n {\left| {\frac{{{x^{(0)}}(i) - {{{\hat{x}}}^{(0)}}(i)}}{{{x^{(0)}}(i)}}} \right| } \end{aligned}$$

6. Construct residual sequence and conduct conditional decision.

If the accuracy is not up to the requirement, the residual tail segment method is used for correction. The residual sequence is defined as:

14 $$\begin{aligned} {\varepsilon ^{(0)}} = ({\varepsilon ^{(0)}}(1),{\varepsilon ^{(0)}}(2),\ldots ,{\varepsilon ^{(0)}}(n)) = ({x^{(0)}}(1) - {{\hat{x}}^{(0)}}(1),{x^{(0)}}(2) - {{\hat{x}}^{(0)}}(2),\ldots ,{x^{(0)}}(n) - {{\hat{x}}^{(0)}}(n)) \end{aligned}$$

If $\forall k \ge {k_0}$, the symbol of ${k_0}$ is the same as that of ${\varepsilon ^{(0)}}(k)$ and $n - {k_0} \ge 4$, then

15 $$\begin{aligned} \left( \left| {{\varepsilon ^{(0)}}({k_0})} \right| \right) , \left( \left| {{\varepsilon ^{(0)}}({k_0} + 1)} \right| \right) ,\ldots ,\left( \left| {{\varepsilon ^{(0)}}(n)} \right| \right) \end{aligned}$$

is modellable residual segment, which can be constructed according to steps (1) to (4). Its response time after the reduced reduction correction is:

16 $$\begin{aligned} {{\hat{x}}^{(0)}}(k + 1) = \left\{ \begin{array}{ll} (1 - {e^a}) \left( {x^{(0)}}(1) - \frac{b}{a}\right) {e^{ - ak}}, &{} \quad k < {k_0}\\ (1 - {e^a})\left( {x^{(0)}}(1) - \frac{b}{a}\right) {e^{ - ak}}\pm {a_\theta }\left( {\varepsilon ^{(0)}}({k_0}) - \frac{{{b_\theta }}}{{{a_\theta }}}\right) {e^{ - {a_\theta }(k - {k_0})}}, &{}\quad k \ge {k_0} \\ \end{array} \right. \end{aligned}$$

That is, the analog value of the G M (1, 1) model is used as the prediction result before ${k_0}$, and the simulated value after the residual segment compensation is used as the prediction result after ${k_0}$. Then perform the error test again and put it into use.

## BP artificial neural algorithm and thinking evolutionary algorithm based on population evolution entropy

### BP artificial neural algorithm

Artificial neural network can simulate human brain to guide the process some highly complex nonlinear problems through simple algorithms. It is a complex network system that reflects the essential characteristics of human brain^30^. Artificial neural network has the characteristics of distributed storage, parallelism and adaptability, etc. which can effectively deal with some uncertain and ambiguous problems, because it is an operational model^31^. BP neural network is the most widely used artificial neural network, which mainly trained by error back propagation, usually descent method^32^, i.e., the correction is completed in accordance with the direction of the negative gradient of the error function. It includes the following two directions: (a) Signal forward propagation. The sample data enters the input layer, passes through the hidden layer, and outputs the result at the output layer. The output data is compared with the expected value, and if the expected value is met, end the process. Otherwise, it will be returned in the reverse direction. (b) Error back propagation. If the output value greatly differentiates from the expected value, the error signal would propagate backwards, from the output layer to the hidden layer to the input layer, the value and threshold are continuously modified during the propagation process to minimize the error value.

Taking the three-layer BP neural network as an example, the input vector is $X = {({x_1},{x_2}\ldots ,{x_i},\ldots {x_n})^T}$, the hidden layer input vector is $Y = {({y_1},{y_2}\ldots ,{y_j},\ldots {y_m})^T}$, the output layer output vector is $o = {({o_1},{o_2}\ldots ,{o_k},\ldots {o_l})^T}$, the expected output vector is $D = {({d_1},{d_2}\ldots ,{d_j},\ldots {d_l})^T}$, the value matrix of the training sample from the input layer to the hidden layer is $V = {({v_1},{v_2}\ldots ,{v_j},\ldots {v_m})^T}$, wherein ${v_j}$ represents the neuron value vector of j in the hidden layer, the value matrix of hidden layer to the output layer is $W = {({w_1},{w_2}\ldots ,{w_k},\ldots {w_l})^T}$, in which ${w_k}$ represents the neuron value vector of *k* in output layer .

For the hidden layer:

17 $$\begin{aligned} \left\{ {_{{h_j} = \sum \limits _{i = 0}^n {{v_{ij}}{x_i}, \quad j = 1,2,\ldots m} }^{{y_j} = f({h_j}), \quad j = 1,2,\ldots m}} \right. \end{aligned}$$

For the output layer to satisfy:

18 $$\begin{aligned} \left\{ {_{{h_k} = \sum \limits _{i = 0}^m {{w_{jk}}{x_j}, \quad k = 1,2,\ldots l} }^{{o_k} = f({h_k}), \quad k = 1,2,\ldots l}} \right. \end{aligned}$$

The activation function of each layer can be a unipolar, bipolar Sigmoid or linear function. For a three-layer BP artificial neural network (one input layer, one hidden layer, and one output layer), its algorithm process is:

1. Initialization. Initialize the value matrix *W* and *V* , assign them with random numbers. The learning rate $\eta \in (0,1)$ , and training precision ${E_{\min }}$ is positive decimal.
2. Input *P* training sample ${X^P}$, calculate each component of *Y* and *O*.
3. Calculate the network error.
  19 $$\begin{aligned} E = \frac{1}{2}\sum \limits _{k = 1}^l {{{({d_k} - {o_k})}^2}} \end{aligned}$$
4. Check the error signal $\delta _k^o$ and $\delta _j^y$ of each layer.
  20 $$\begin{aligned} \delta _k^o= & {} ({d_k} - {o_k}){o_k}(1 - {o_k}) \end{aligned}$$
  21 $$\begin{aligned} \delta _j^y= & {} \left( \sum \limits _{k = 1}^l {\delta _k^o{w_{jk}}} \right) {y_j}(1 - {y_j}) \end{aligned}$$
5. Adjust the values of each layer, calculate each component of *W* and *V*.
  22 $$\begin{aligned}&\Delta {w_{jk}} = \eta ({d_k} - {o_k}){o_k}(1 - {o_k}){y_j} \end{aligned}$$
  23 $$\begin{aligned}&\Delta {v_{ij}} = \eta \left( \sum \limits _{k = 1}^l \delta _k^o{w_{jk}}\right) {y_j}(1 - y){x_i} \end{aligned}$$
  24 $$\begin{aligned}&\left\{ {_{{{v'}_{ij}} = {v_{ij}} + \Delta {v_{ij}}}^{{{w'}_{jk}} = {w_{jk}} + \Delta {w_{jk}}}} \right. \end{aligned}$$
6. Check whether the training process can be ended, including the times of completed training and accuracy requirements.
7. Make predictions.

### Populational entropy based mind evolutionary algorithm (PEMEA)

Mind Evolutionary Algorithm (MEA) is an evolutionary algorithm simulating the human mind evolutionary progress, which mainly uses convergence and alienation to replace the individual optimization by group optimization and avoid the defects of genetic algorithm. The convergence operation is the process of becoming the winner through individual competition within the group, while the alienation operation is the process of sub-group competition. This process ensures that new points are continuously explored throughout the solution space. Convergence and alienation operations are iteratively performed until the algorithm termination condition is met. The positive feedback mechanism in MEA is conducive to the development of the group to survive, the negative feedback mechanism to prevent the algorithm premature and avoid falling into the local optimal solution. Structurally speaking, the parallelism of MEA has high search efficiency and is also exceedingly robust to interference^33^.

PEMEA introduces the thermodynamic entropy concept to form an entropy based sampling optimization method to estimate the search space, making exploration more adequate and more purposeful^34^. When solving the optimization problem, the MEA algorithm first randomly generates the initial population of the scale N in the solution space, performs local search in the vicinity of each individual through the convergence operation, and then a new individual is generated globally by the alienation operation to supplement the eliminated partial solution. As evolution progresses, population progress gradually approaches the optimal solution. The population evolution entropy characterizes the degree to which the population is close to the optimal solution. At the beginning of evolution, the distribution is completely random, and the optimal solution is completely undetermined. At this time, the entropy value *H* is the largest. As the evolution progresses, the population gradually grasps the change of the optimal solution, and slowly determines it until it is completely affirmed. From the change of entropy value, the higher the position of the optimal solution is, the smaller the search range of the algorithm is. The efficiency of the algorithm becomes higher and the entropy value *H* gradually reduces. When the population is already distributed in the limited range of optimal solution, the entropy value *H* reaches a minimum. In the PEMEA algorithm, the alienation operation ensures the global traversal of the population, avoiding local optimization, and the convergence operation achieves the task of “fine search”. Therefore, linking the change of evolutionary entropy with the convergence search width will improve the searching efficiency of MEA.

Set ${\sigma _i}$ as the *i* generation of convergence variance, *M* as population size, ${m_i}$ represents the individual in the population which is assigned to the interval *i*, ${{\hat{p}}_i}$ represents the probability that an individual appears in interval *i*. ${{\hat{H}}_P}(i)$ is the evolutionary entropy estimate of the *i* generation population, and the relationship between ${\sigma _i}$ and ${{\hat{H}}_P}(i)$ is expressed as follows:

25 $$\begin{aligned} \sigma _i^2= & {} C \cdot {{\hat{H}}_P}(i) \end{aligned}$$

26 $$\begin{aligned} C= & {} \frac{1}{{\ln M}} \end{aligned}$$

Evolutionary entropy as:

27 $$\begin{aligned} {{\hat{H}}_P}(i)= & {} - \sum \limits _{i = 1}^r {{{{\hat{p}}}_i}\ln {{{\hat{p}}}_i}} \end{aligned}$$

28 $$\begin{aligned} {{\hat{p}}_i}= & {} \frac{{{m_i}}}{M}(i = 1,2,\ldots ,r) \end{aligned}$$

The steps of the PEMEA algorithm are as follows:

1. Initialization. The initial population is randomly generated in the solution space, and the score is calculated. The score function is the reciprocal of the mean square error of the sample. Select $N_S$ highest individuals to form the winner sub-population, publish it on the global bulletin board and sort them, and the rest are temporary sub-populations, which are randomly distributed to form $N_T$ temporary sub-populations.
2. Convergence. The convergence operation is carried out in the above two sub-populations, and the progeny individuals are randomly generated centering on the parent sub-population, and the production process is as shown in (29). Then, the local search and competition are conducted among the winners to generate the new winners are generated. The individual information will be published on the local bulletin board, and the score of each sub-population winner is the score of the sub-population.
  29 $$\begin{aligned} {x^k} = N({x^{k - 1}},{\sigma ^k}) = {x^{k - 1}} + {\sigma ^k} \cdot r \end{aligned}$$
  wherein *r* is the random number generated by the standard normal distribution *N*(0, 1) . Equations (25) to (29) indicate that as the entropy value becomes smaller and smaller, the search width becomes smaller and smaller, the search accuracy would become finer and finer, and the search efficiency also increases.
3. Alienation. The alienation operation is carried out in a global scope, and the sub-populations with low scores are replaced by sub-populations with high scores, and the sub-populations that are replaced are redistributed in space to form new temporary sub-populations.
4. Judgment convergence. If the conditions to end the process is not reached, repeat (25) to (29).

## Residual GM network model based on BP-PEMEA correction

### Reasons behind BP-PEMEA

Since its inception, GM has become essential for a wide range of fields and achieved considerable promising application results. But it is not perfect. The biggest challenges are its large prediction bias and narrow scope of application^35^. The modellable residual tail segment can effectively correct GM to improve the prediction accuracy, but it strictly limited the conditions that at least more than 4 tail segments should have the same residual sign. Geometrically speaking, the whole simulated sequence tail segment must be adjusted above or below the actual sequence segment and to be corrected through providing reverse adjustment. However, such a situation is not shared in practical applications; most practical situations present the feature of volatility or alternation, which can greatly limit the application of GM.

This paper includes BP artificial neural network to modify the tail segment mainly due to its three characteristics: (a) Its capability of simulating a variety of functions, such as nonlinear functions, piecewise functions, etc. (b) It does not require to assume in advance that there is a certain type of functional relationship between the data, and the artificial neural network can establish the correlation relationship according to the provided training data and its own attributes without designing the parameter distribution in advance. (c) It can realize high information utilization and effectively avoid the loss of information triggered by positive and negative offset in the data processing process. Therefore, the artificial neural network is particularly suitable for residual correction of the GM (1, 1) model and effectively provide the required “supplemental amount”. However, BP artificial neural network also has its own shortcomings, the most crucial drawbacks are: (a) The learning speed of convergence is slow. Gradient descent method is the core of BP algorithm. Because of the complex and variable nonlinearity of its processing, the appearance of “zigzag phenomenon” is inevitable. In the flat area of the error surface, the error gradient will have some small changes. Even with a larger value adjustment, the error reduction is still slow. (b) It is easy to get a local minimum. After the initial value is given to the network, the value is adjusted according to the forward direction and error reversed. If the initial value is improperly assigned, it may fall into a local minimum. Therefore, the BP algorithm needs to be further modified. According to the current mainstream algorithms, the BP model based on genetic algorithm (GA-BP) has higher accuracy, but its convergence speed is slower, and it is not guaranteed to obtain the global maximum. This is because the GA algorithm’s crossover and mutation operations can produce both good genes and inferior disruptive genes, indicating that evolution is not oriented in genetic algorithms. To solve the above-mentioned problems, we use PEMEA to amend the BP artificial neural networks. Unlike genetic algorithms, MEA records the competition information of individual and sub-populations for each iteration, allowing evolution to move in a favorable direction. In other words, evolution is directional in MEA. Meanwhile, the convergence and dissimilation operations in MEA algorithm represent the selection within the sub-population and the whole group respectively, and the two processes are developed in parallel to improve the global search efficiency of the whole system. On the basis of MEA, the meaning of information entropy is introduced, that is, with the development of evolution, the position change of the population to the optimal solution is determined from complete uncertainty to final affirmation and the search range becomes smaller and more detailed. Linking the change in information entropy to the search width also further improves search efficiency^36–38^.

### The procedure of residual GM based on PEMEA-BP correction

After PEMEA algorithm output the optimal connection value to the BP artificial neural network, the residual BP sequence will be simulated by a trained BP artificial neural network to provide the “replenishment amount” required for the entire GM model. In this way, it can not only have the advantages of GM to simulate small samples with high precision, but also expand the scope of use to various nonlinear or even multi-dimensional objects. Moreover, it can avoid the defects that BP artificial neural network converges slowly and is easy to fall into local minimum. The specific steps are as follows:

1. Establish a system behavior time series ${X^{(0)}}$ according to (1) and (2) and then generate a 1-AGO sequence ${X^{(1)}}$ and an immediate sequence ${Z^{(1)}}$.The construction of these two sequences is a param feature of GM, which aims to further uncover the information inherent in these seemingly erratic data.
2. Using the least squares estimation to obtain the parameter vector ${\hat{a}}$ according to (3) to (8), the obtained time response equation ${X^{(0)}}$ and the subtractive reduction equation ${{\hat{x}}^{(0)}}(k)$, then establish a simulation sequence.If the requirement for simulation accuracy is low, a preliminary simulation can be performed at this time, but the simulation value gradually increases with the prediction time.
3. Calculate the error and use it if the accuracy meets the requirements according to (9) to (11); otherwise, establish a residual sequence ${\varepsilon ^{(0)}}$ in accordance with (13). Use the residual GM method if the residual tail segment modeling condition is met. If not, normalize the residual sequence.The purpose of data normalization is to allow pre-processed data to be confined to a certain range, thus eliminating the effects of differences in magnitude, etc.
4. Use the residual sequence to generate the test set and training set, determine the numbers of input layer, hidden layer and output layer nodes, and construct the topology of BP artificial neural network.
5. Encode the network connection value, determine the score function, and generate the initial population according to the score function. Rank the individuals according to the score, and the individuals with high scores are taken as the center, and new individuals are generated in the vicinity of the individual to form the superior subpopulation and the temporary sub-populations; and publish them on the global bulletin board.
6. Perform a convergence operation in the sub-populations. Determine whether each sub-population is mature, and if true, end the convergence; otherwise, generate progeny population around the parent according to the Eq. (29) and continue the convergent operation until the sub-population matures. Each individual information is announced on a local bulletin board; and set the highest score of each sub-population as its group score.
7. Perform an alienation operation in the global scope. If the temporary sub-population score is higher than the matured sub-population, then replaced it. Then redistribute the individuals within the replaced sub-population in space to form new sub-populations; otherwise, release the temporary sub-population.
8. Determine the convergence. If it converges or reaches the end condition, proceeds to step (9), otherwise returns to step (6).
9. Decode the optimal individual and set it as the value of BP artificial neural network. Complete the training of BP network by using the test set and expected value in step (4).
10. Predicate residual sequence $\{ {\varepsilon ^{(0)}}(L)\}$ by the trained BP network and construct a new simulation sequence ${{\hat{x}}^{(0)}}(i,1)$ based on it, ${{\hat{x}}^{(0)}}(i,1) = {{\hat{x}}^{(0)}}(i) + {\varepsilon ^{(0)}}(i)$ . The simulated sequence is the predicted value of the combined model.
11. Calculate the error accuracy.

**Figure 1:**
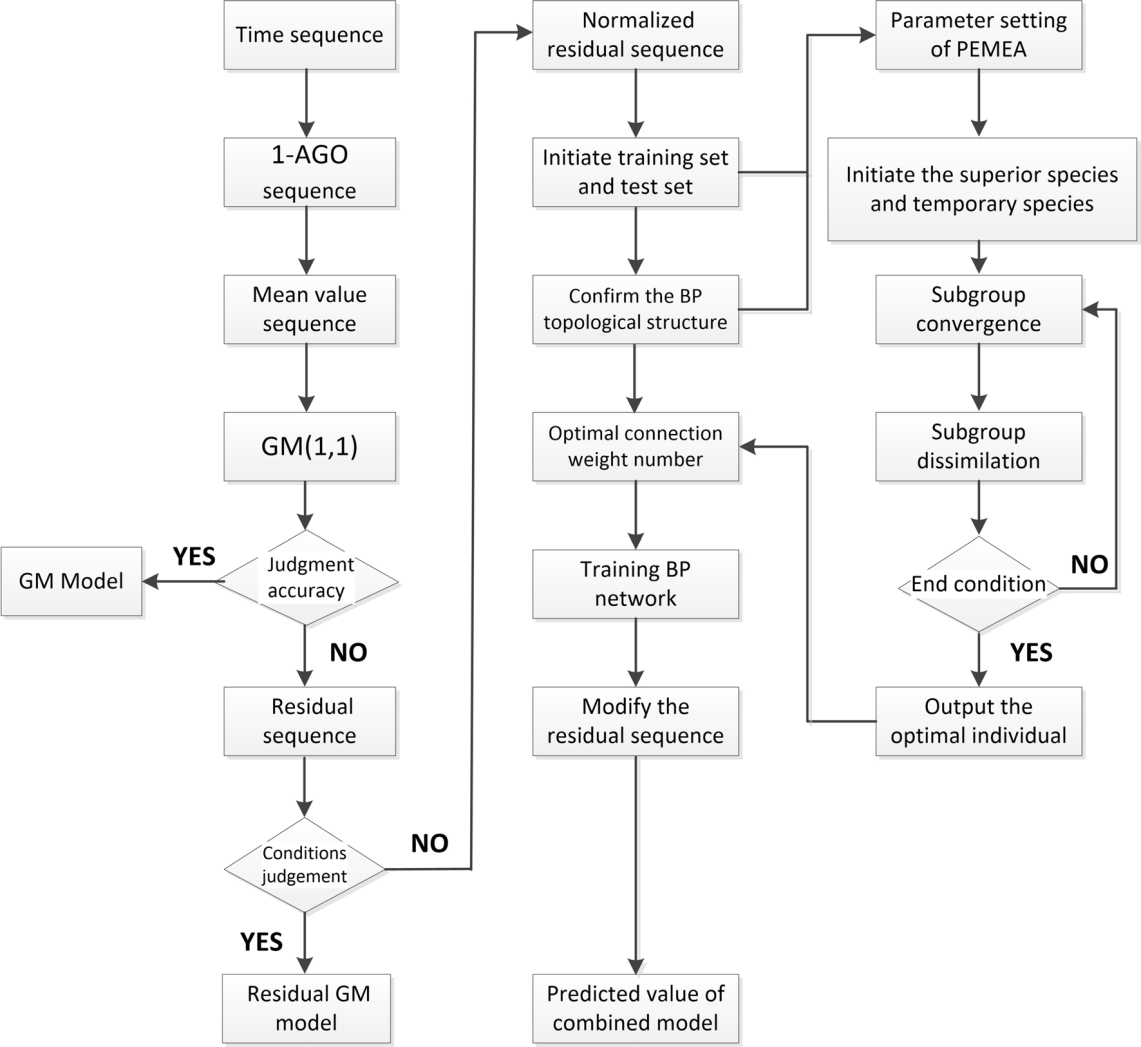
PEMEA-MP modified GM residual model flow chart.

The PEMEA-MP modified GM residual model flow chart is shown in Fig. 1.

## Empirical study

### Parameter determination

Compared with the grey prediction model, the artificial neural network has a powerful learning ability. It can learn from the predictable mutation data to predict the occurrence of some special situations. However, artificial neural networks also have their weaknesses, that is, they require a large amount of widely representative training data, which is difficult to provide in actual practice. And the untrained artificial neural network will have large margin of error when making predictions. If the neural network prediction completely replaces the grey prediction, then the smooth data that accounts for most of the data must be trained. Too many training modes will necessarily require a larger network structure, which reduces the learning efficiency and consumes considerable resources, and the learning of a few special data points may also be overwhelmed by a large amount of normal data, which makes it not prominent enough. Neural network prediction is not suitable for process prediction with stable processes but sudden change points^38–43^.

#### Data training and input layer determination

Set ${e^{(0)}}(L)$ as residual sequence. If S is the prediction order, the input samples for network training are ${e^{(0)}}(i - 1)$ ,${e^{(0)}}(i - 2)$,$\ldots$,${e^{(0)}}(1 - S)$,and ${e^{(0)}}(i)$ is the corresponding output samples of the network, where i = 1,2,..., n. Here, the prediction order is 3, which means that the number of network input layers is 3.

#### Data volume determination

Randomly generate 10, 20, 30, 40, 50, 60, 70 data, and fit the BP, GENETIC-BP, and PEMEA-BP algorithms to obtain the mean square error as shown in the following Table 1.

**Table 1:** Data volume-MSE.

Data volume/MSE	BP	GENETIC-BP	PEMEA-BP
10	4065	7.5852	15.7611
20	5467	6014.4	1.6113
30	6774	0.0004375	0.3154
40	104,480	0.5994	0.0252
50	444,480	0.0061	0.0484
60	514,480	0.000034971	0.0727
70	644,480	0.0018	0.0574

**Figure 2:**
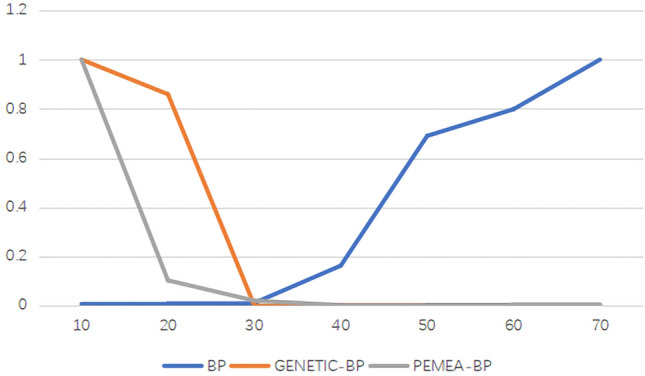
Data volume mean square error.

To be more intuitive, the above values are normalized and displayed in the following Fig. 2.

Although the grey prediction model studies “some information is known and some information is unknown” “small sample” and “poor information” uncertain systems, if you want to optimize the grey prediction model by neural network, you need to determine the minimum data size. As can be seen from the Table 1 and Fig. 2, BP can converge when the amount of data is small, and it is difficult to converge to the global optimal value when the amount of data is increased. GENETIC, PEMEA is difficult to converge to the optimal value when the amount of data is small, the amount of data is increased, and the accuracy is gradually accurate. As can be seen from the figure above, when the amount of data reaches 50, the error of the BP algorithm increases significantly. Therefore, the number of residual tails in this experiment is 40.

#### Determination of other parameters

The number of hidden nodes in the network is usually determined according to the formula ${n_1} = \sqrt{n + m} + a$ or ${n_1} = {\log _2}n$ , where m is the number of output neurons and n is the number of input units. The hidden node is set to 8. The activation functions between the input layer and the hidden layer, and between the hidden layer and the output layer of the BP neural network are sigmoid functions. The hidden layer uses the tansig transfer function and the output layer uses the logsig transfer function. The maximum training times: 50,000 learning rate: 0.005 target. Error: le−3. Number of GENETIC iterations: 200. Population size: 10. Crossover probability: 0.4. Mutation probability: 0.2. The PEMEA algorithm takes a population size of 200, the number of winning subpopulations is 5, the number of temporary subpopulations is 5, and the number of iterations is 10.

### Algorithm verification

#### Data source

This article select the number of confirmed cases of COVID-19 in South Africa from June 9 to July 18 in 2020 (Coronavirus disease Weekly Epidemiological Update and Weekly Operational Update. https://www.who.int/emergencies/diseaes/novel-coronavirus-2019/situation-reports/) as the training data of the residual tail in Table 2, and the number of confirmed cases from July 19 to July 31 as the test data of residual tail in Table 3.

**Table 2:** Training set data.

Date	6.9	6.10	6.11	6.12	6.13	6.14	6.15	6.16	6.17	6.18
	50,879	52,991	55,421	58,568	61,927	65,736	70,038	73,533	76,334	80,412
Date	6.19	6.20	6.21	6.22	6.23	6.24	6.25	6.26	6.27	6.28
	83,890	87,715	92,681	97,302	101,590	106,108	111,796	1,118,375	124,590	131,800
Date	6.29	6.30	7.1	7.2	7.3	7.4	7.5	7.6	7.7	7.8
	138,134	144,264	151,209	159,333	168,061	177,124	187,977	196,750	205,721	215,855
Date	7.9	7.10	7.11	7.12	7.13	7.14	7.15	7.16	7.17	7.18
	350,879	364,328	373,628	381,798	394,948	408,052	421,996	434,200	445,433	452,530

**Table 3:** Testing set data.

Date	7.19	7.20	7.21	7.22	7.23	7.24	7.25	7.26	7.27	7.28
	350,879	364,328	373,628	381,798	394,948	408,052	421,996	434,200	445,433	452,530

**Figure 3:**
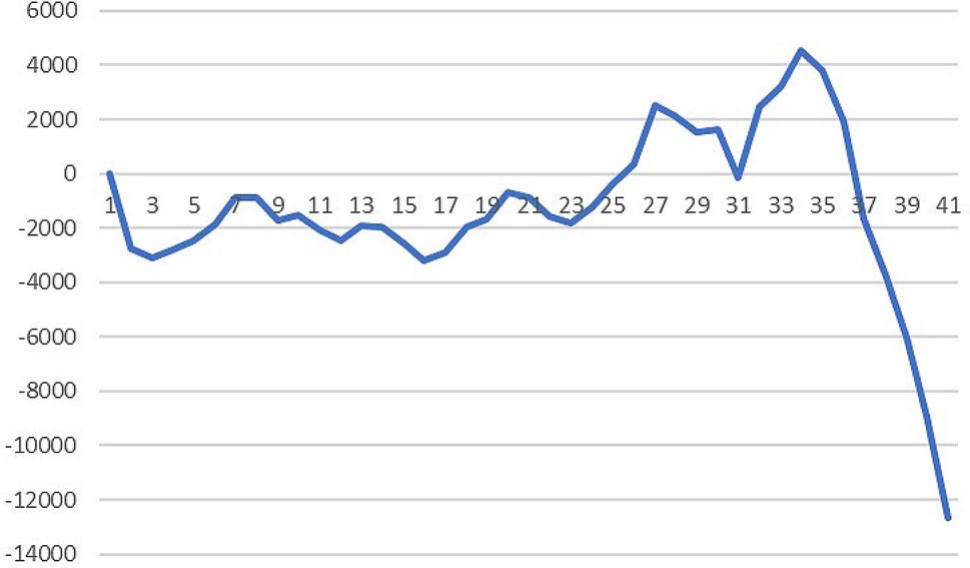
Residual of GM (1,1) simulation sequence.

According to Eqs. (4) to (8), a$$ = -$$ 0.0513 and b = 51434. The simulation diagram is shown in Fig. 3. From the simulation, the average simulation relative error of the original model is 1.0224%, the error is too high, and the residual sequence $$ {\varepsilon ^{(0)}} $$ does not meet the modeling conditions of the tail section.

#### Fitting accuracy

**Figure 4:**
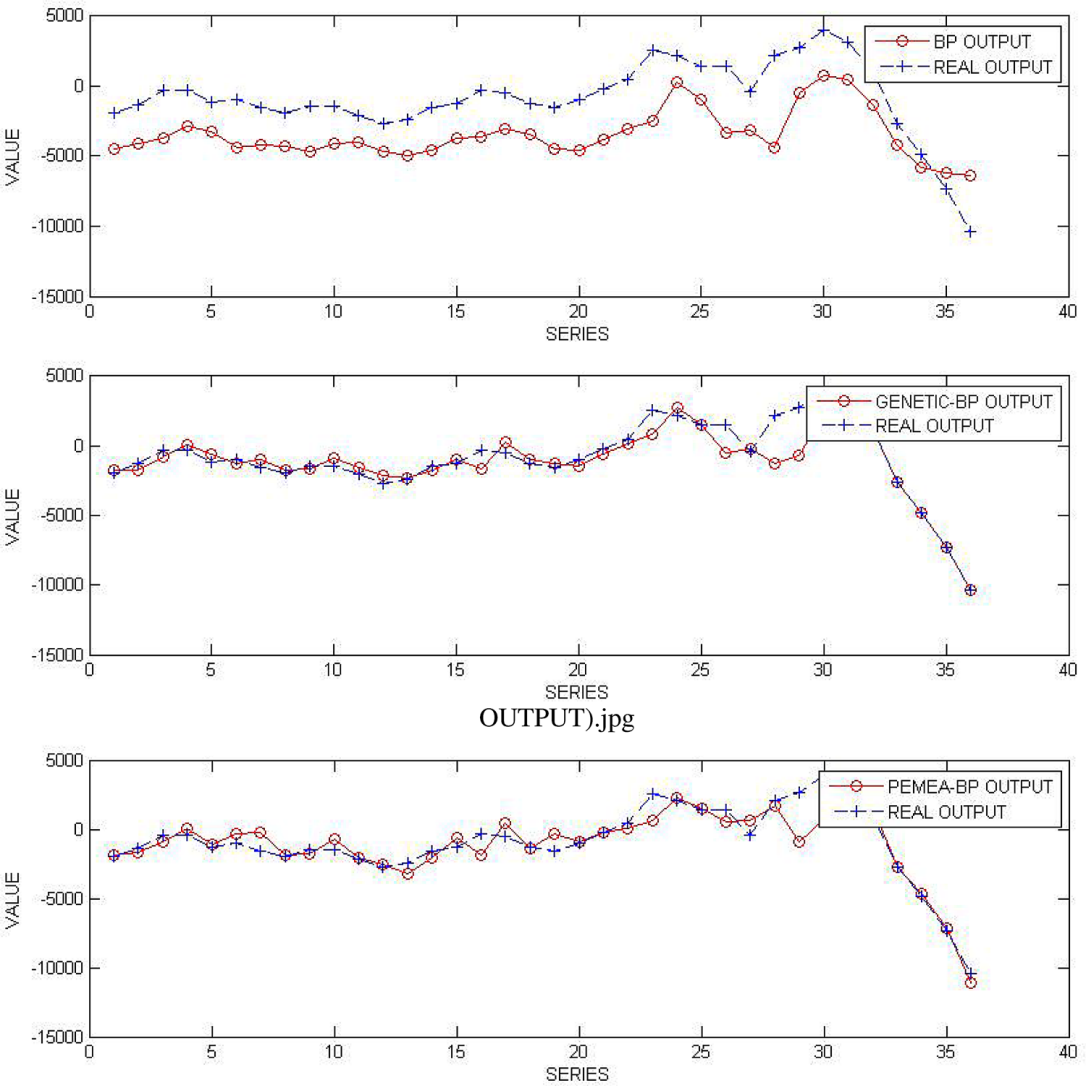
Comparison of actual data and network output.

The actual data and network output data are shown in Fig. 4.

**Table 4:** Fitting accuracy table.

	SSE	MAE	MSE	MAPE	MRE	TIME
BP	1043019.411	153.200	28972.761	0.014	$-$ 0.022	2.022
GENETIC-BP	96314.456	41.518	2675.402	0.004	$-$ 0.001	168.594
PEMEA-BP	41573.411	27.701	1154.817	0.002	$-$ 0.002	1.505

The fitting accuracy table is shown in Table 4.

**Figure 5:**
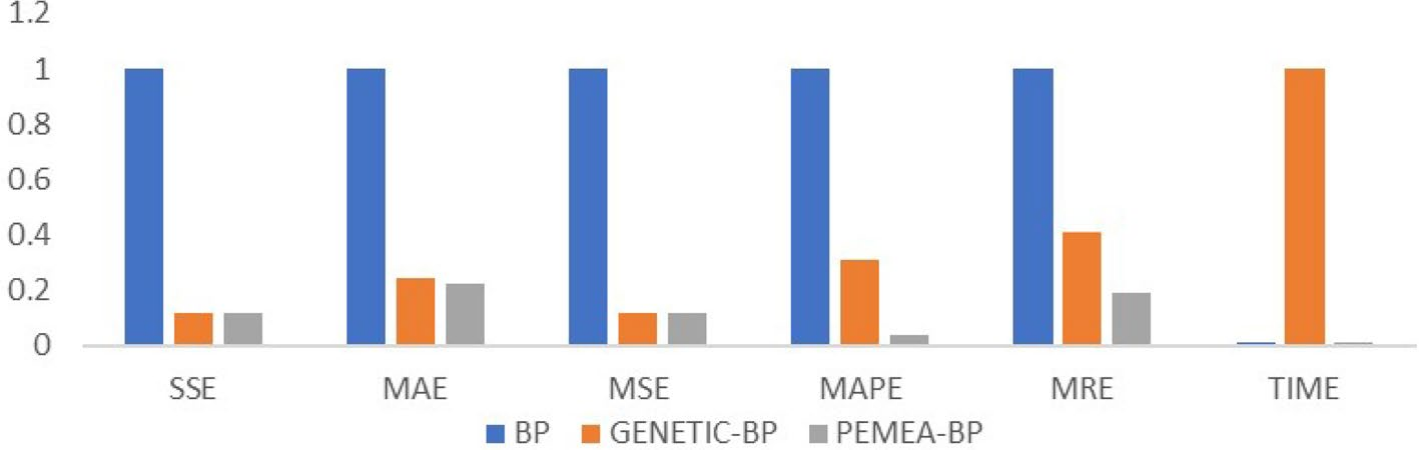
Normalized fitting accuracy chart.

To be more intuitive, the above values are normalized and displayed in Fig. 5.

It can be seen from Table 4 and Fig. 5 that PEMEA-BP has significantly improved in time and accuracy compared with BP and GENETIC-BP.This model aims at the situation that there are many uncertain factors in small samples, so there are still some relative errors. It is more suitable for a macro relatively accurate prediction. However, when it comes to a specific point, it is still neural network and ordinary differential equation with known variables that are more accurate.

#### Prediction accuracy

The residual sequence predicted by the BP network training model is $\{ {{\hat{e}}^{(0)}}(L)\}$, and the predicted value constructed using this predicted value is ${{\hat{x}}^{(0)}}(i,1) = {{\hat{x}}^{(0)}}(i) + {{\hat{e}}^{(0)}}(i)$. The predicted value ${{\hat{x}}^{(0)}}(i,1)$ is obtained by the combination model of grey and neural network.The forecast accuracy table is shown in Table 5.

**Table 5:** Forecast accuracy table.

	SSE	MAE	MSE	MAPE	MRE
GM	5,018,968.753	185.215	139,415.799	0.008	0.008
BP+GM	1,424,263.499	92.917	39,562.875	0.004	0.004
GENETIC-BP+GM	133,421.416	29.532	3706.150	0.001	0.001
PEMEA-BP+GM	74,868.939	18.717	2079.693	0.001	0.001

**Figure 6:**
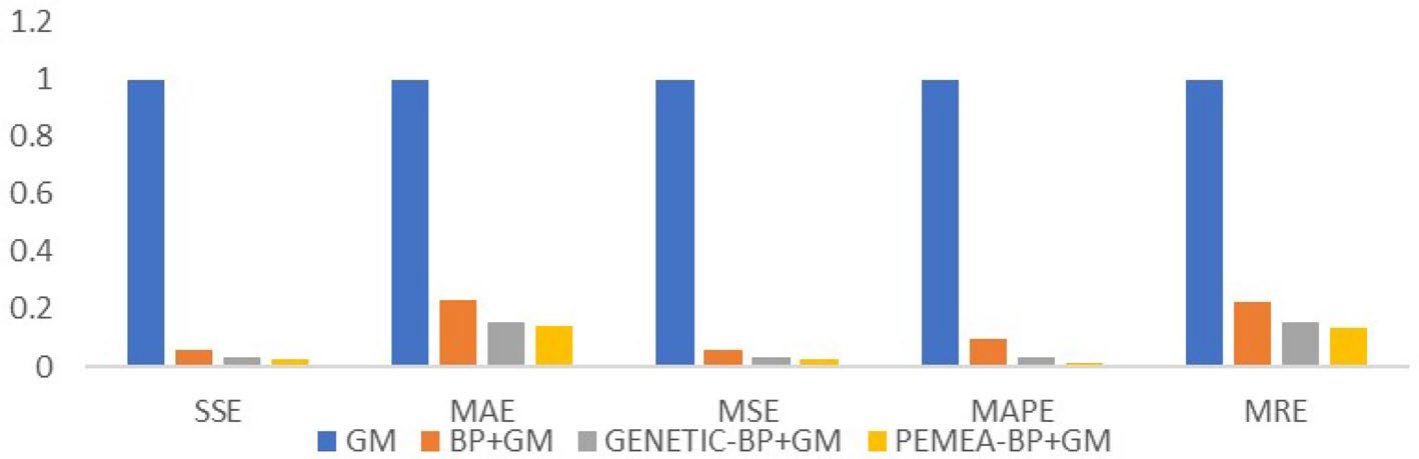
Normalized prediction accuracy chart.

To be more intuitive, the above values are normalized and displayed in Fig. 6.

From Table 5 and Fig. 6, it can be seen that the residual GM modified by PEMEA-BP greatly compensates for the lack of accuracy of GM.The accuracy and BP convergence rate of GM in the case of inconsistent residual tail symbols are effectively compensated.

## Conclusion

The GM method is widely used because it can effectively solve the problem of “less data” and “poor information”, but it has high requirements on the input data, that is, the input tail data should be linear and bear the same sign. However, in reality, More sequences present waveform or highly non-linear features. If these characteristics are not met before input data, the prediction accuracy will be low. The algorithm proposed in this paper uses the residual tail as a sample to input the trained BP artificial neural network model, which can obtain an effective residual sequence prediction value ${{\hat{\varepsilon }} ^{(0)}}$. BP artificial neural network has the characteristics of being easy to fall into a local minimum and slow convergence. This is corrected by PEMEA to output the best connection weight. Finally, the residual grey model is used to obtain the final predicted value. The residual grey model modified by PEMEA-BP not only has the advantages of GM, i.e. small samples and high accuracy, but also can expand the scope of use to various non-linear and even multidimensional objects. Meanwhile, it can avoid defects of other algorithms, such as slow convergence and easy to fall into the local minimum. In small sample data experiments, judging from SSE, MAE, MSE, MAPE, MRE and other indicators, this new algorithm has significant advantage over GM, BP algorithm and combined genetic algorithm in terms of simulation accuracy and convergence speed.
